# The role of self-perception in mental health: current insights on self-esteem and self-schemas

**DOI:** 10.3389/fpsyt.2025.1683194

**Published:** 2025-12-12

**Authors:** Radhwan Hussein Ibrahim, Mariwan Qadir Hamarash, Abdulhakeem Jamil Ahmed, Salwa Hazim Al Mukhtar, Marghoob Hussein Yaas

**Affiliations:** 1 Department of Clinical Nursing Sciences, College of Nursing, Ninevah University, Mosul, Iraq; 2 College of Nursing, AL Kitab University, Kirkuk, Iraq; 3 Department of Nursing, Mosul Medical Technical Institute Northern Technical University, Mosul, Iraq; 4 Department of Clinical Nursing Sciences, College of Nursing, University of Mosul, Mosul, Iraq

**Keywords:** self-perception, self-esteem, self-schemas, mental health, Iraq, post-conflict, adults

## Abstract

**Background:**

Self perception includes both self-esteem and self-schema and is an important factor in determining an individual's mental health. This is particularly true in contexts that have been affected by war or conflict, such as post-conflict Mosul, Iraq. The city of Mosul has experienced long term exposure to trauma, displacement and social economic hardship; these experiences will likely cause disruption to the personal and community identities of residents. Therefore, it is necessary to understand how self-perception relates to mental health in order to develop effective interventions.

**Aim:**

This research aims to investigate the relationship between self-perception (self-esteem and self-schema) and mental health in adults living in Mosul. Additionally, we aim to identify cultural and context factors which may influence the relationship between self-perception and mental health.

**Methods:**

Using a cross-sectional mixed-methods design, 232 adults who live in Mosul participated in our research. We used three quantitative instruments: the Rosenberg Self Esteem Scale, the Self Schema Questionnaire, and the General Health Questionnaire-28 to collect quantitative data. The qualitative data were collected via 20 semi-structured interviews with participants who were purposefully selected from our sample of 232. Descriptive statistics, Pearson correlation and multiple linear regression were used to analyze quantitative data. Thematic analysis was used to analyze qualitative data.

**Results:**

Low self-esteem was reported by 41.4% of participants, and over half (52.6%) showed signs of possible psychological distress. Self-esteem (r = 0.52, p < 0.001) and positive self-schemas (r = 0.48, p < 0.001) were positively correlated with mental health, while negative self-schemas were negatively correlated (r = –0.55, p < 0.001). Multiple regression identified self-esteem (β = 0.37, p < 0.001), negative self-schemas (β = –0.34, p < 0.001), and employment status (β = 0.19, p = 0.014) as significant predictors of mental health, explaining 46.2% of the variance. Qualitative findings revealed three themes: identity reconstruction after conflict, cultural pressures on self-perception, and resilience through community support.

**Conclusion:**

Perceptions of oneself are an important predictor of mental health in adults living in Mosul, Iraq. Enhancing positive self-esteem and reducing maladaptive self schemas may increase psychological resilience in post-conflict settings. Culturally sensitive interventions that incorporate community and family support systems are recommended.

## 1 Introduction

There is a growing recognition that mental health is a significant contributor to our overall wellbeing, influencing our emotional and cognitive functioning, our ability to interact and form relationships with other individuals, and ultimately our quality of life (1, 2). Among the multitude of psychological constructs that are associated with mental health, self-perception, which includes self-esteem and self-schemata, is critical in framing how we interpret experiences and influence how we encounter stress and socialization (3). Self-perception is the mental image or concept that a person has about themselves and typically is a combination of personal experiences with cultural norms and social interaction (4). When the self-perception is positive and realistic, it can serve as a buffer against psychological distress, while negative or poorly formed self-perceptions can add risk of vulnerability to developing psychological disorders such as depression, anxiety, or maladaptive coping (5).

Self-esteem is the most important part of self-perception, as it is the value and acceptance of yourself (6, 7). Self-esteem consistently differentiates emotional control, absent maladaptive coping or resilience to life's stressors, as self-esteem can foster more successful emotional regulation and adaptive coping behaviors, while low self-esteem can predispose to mood disorders, avoidance strategies (withdrawal), and self-stigmatizing behaviors (8, 9). Self-schemata are cognitive generalizations about the self based on past experiences and operate like organizing principles, and facilitate computations when we process and categorize information, recall memories, and imagine future potential (10, 11). Schemata can be healthy and adaptive if they remain grounded in reality, while maladaptive self-schemata are often formed during chronic stress and trauma and consist of reinforcement through thought and are often unaddressable in terms of psychological recovery (12). Mosul, Iraq, is a compelling context for understanding the impact of self-perception on mental health (13–15). Mosul has undergone many years of social-political change, armed conflict, migration, and interruption of economic status resulting from the previous twenty years, and will have many effects on the mental health of Mosuleans (16–18). Exposure to prolonged stressors such as war related trauma, job loss, separation from loved ones, and lack of mental health service availability, all contribute to the experience of high levels of distress among the people of Mosul. In this case, self-perception is likely to represent both a protective factor and a risk factor, as self-perception will reflect how individuals manage with the difficulties of living in war-time, how they re-establish or re-formulate their sense of identity and how they re-engage in community life (19, 20).

The cultural context of self-perception in Mosul is very important (21, 22). Historically, Mosul has been culturally and socially diverse and inter-connected. Thus, the cultural structures of community, family, honor and social standing of Mosul may provide a clear alternative to the stigma associated with mental illness. While the cultural practices of Mosul may provide strong social supports, they may at the same time be contributing to stigma and the internalization of "ugly" affects if someone develops psychological distress. The contrast between the cultural system and self-perception highlights the need for a more detailed examination of how self-concept, self-esteem and self-schemas are developed and function.

Recent developments in Cognitive Theory suggest that the development of self-schemas—lasting cognitive representations of the self based on past experiences—are the foundation of our emotional and behavioral responses throughout life. Furthermore, Schema Therapy provides an integrated theoretical approach for understanding the role of early maladaptive schemas (EMS)—established through the failure to meet one's emotional needs during childhood—in the development of long-term psychological distress and the establishment of an individual's pattern of personality. A recent comprehensive review of the EMSs identified four higher-order domains: disconnection/rejection, impaired autonomy/performance, excessive responsibility/standards, and impaired limits. These domains represent core aspects of human vulnerability and illustrate how adverse early developmental experiences lead to poor mental health outcomes later in adulthood. Examining self-schemas and cognitive distortions in relation to self-esteem, emotional well-being, and psychological resilience—the primary focus of the current study—will allow for a better understanding of the impact of negative self-schemas on an individual's ability to develop positive self-concepts.

These domains represent core dimensions of human vulnerability, linking adverse developmental experiences to adult mental health outcomes. Incorporating this perspective helps clarify how negative self-schemas and cognitive distortions can influence self-esteem, emotional well-being, and overall psychological resilience—key constructs explored in the present study.

Specifically, current literature states that interventions to promote self-perception, such as cognitive-behavioral therapy (CBT) interventions, resilience training, and unique psychosocial and social community programs have a positive effect on mental health, particularly the mental health of communities. Yet, there is a severe lack of research addressing the forms of self-perception of individual in conflict-affected urban populations like Mosul. Moreover, most literature that is available to contextualize self-perception was done in a Western context, which would lead to misconceptions and assumptions to communities that experienced, and continue to try and make sense of socio-political histories. The emergence of context of changing self-esteem and self-schemas in Mosul may have important implications for applying culturally relevant, informed mental health interventions to be utilized when recovering from conflict for public health and planning purposes.

Self-perception has significant implications for mental health care in Mosul. For clinicians, they would assist in assessment screening and developing referral pathways for those who are vulnerable to developing psychological disorders via recognizing self-perception. This aspect should be included in post-conflict mental health planning. For health policy, investing in community education programs and community investment that develops self-perception can address addiction plus other maladaptive self belief systems, thus achieving public mental health goals. For community, developing an awareness of potential cultural systems surrounding self-perception and mental health stigma can destabilize stigma, and encourage developing coping responses via help-seeking behaviors; especially to vulnerable populations whose self-perception are developing resilience.

Studies in other Iraqi regions, such as Baghdad and Basra, have reported that psychological distress and low self-esteem remain prevalent among adults exposed to prolonged instability, with rates ranging from 40–60% (23). Similar findings were observed in Syria (24) and Lebanon (25), where conflict-related trauma significantly impaired self-perception and emotional regulation.

Given the considerations above, this study will measure the relationship between self-perception, specifically self-esteem and self-schemas, and mental health among adults in Mosul. The intention of the research is to embody psychological theory with the realities of the lived experience of Mosul, to contribute to bridging some of the gap between universal mental health knowledge and local realities for people in the city. The contribution of this research will potentially impact the development of culturally sensitive interventions that not only consider psychological symptoms, but also works towards promoting the individuals from resilience to using and transforming back their self-perception to promote community wellness in a city that is beginning to reclaim its identity from the brutality of conflict.

## 2 Materials and methods

### Study design

The cross-sectional study employed a mixed methods design to investigate the relationship between self-perceptions and mental health. Quantitatively, the goal of the quantitative approach was to measure how high or low participants were with respect to self-esteem and self-schemas and also to assess the correlation between facets of self-perception and aspects of mental health; qualitatively, the goal of the qualitative approach was to identify participants' self-perceived experiences of self-perception through participants' personal stories (narratives) as well as participants' culturally-based self-perceived experiences of self-perception (i.e., what participants believe about self-perception based on the culture they live in) to better understand participants' experiences of self-perception within the context of their post-conflict lives.

### Study location and population of interest

The study was carried out in Mosul (the capital city of the Nineveh Province) in Northern Iraq. In excess of two million people live in Mosul which is currently experiencing significant social, economic and infrastructure problems due to many years of conflict. The targeted population included all adults 18 years and older who have lived in Mosul for at least 2 years and represent a variety of educational backgrounds, social status, occupations, and economic statuses. All individuals meeting the demographic characteristics outlined and able to provide an informed response were eligible to take part in the research. However, all participants who experienced a condition(s) that would have significantly impaired their cognitive functioning or ability to accurately report on themselves, such as being severely cognitively disoriented, having chronic neurological disorder(s) that affected their level of consciousness, or being in a current major psychiatric crisis were excluded from this study.

### Sample selection and sample size

In Mosul, to allow for representation from each of the different districts, including districts in central urban Mosul and peripheral neighborhoods, a multistage stratified random sampling design was used. For the quantitative method, GPower was used for the analysis, assuming a medium effect size (f² = 0.15), with 95% confidence level and 80% power required resulted in the need for a sample size of a minimum 200 as per the statistical analysis. To account for potential non-respondents, or incomplete surveys, we aimed to recruit 240 participants. For the qualitative component we purposively sampled from the quantitative sample, where we will purposefully select 20 participants for additional in-depth interviews to learn about individual perspectives in a more maximum depth fashion. We will include gender, age and socio-economic status for variations purposes to allow for maximum variation.

### Data collection tools

There were two major types of tools:

#### Quantitative tools

##### Rosenberg Self-Esteem Scale

The Rosenberg Self-Esteem Scale (RSES), originally developed by Rosenberg (1965) (26), is a 10-item instrument designed to measure global self-worth by assessing both positive and negative feelings about the self. Each item is rated on a 4-point Likert scale ranging from *1 (Strongly Disagree)* to *4 (Strongly Agree)*, with total scores ranging from 10 to 40. Higher scores indicate greater self-esteem, while lower scores reflect low self-esteem or self-doubt. A total score below 25 is generally interpreted as indicating low self-esteem, 26–29 as moderate, and 30 or above as high self-esteem. The Arabic version of the RSES has demonstrated strong psychometric properties and cross-cultural validity in Arab populations (Cronbach’s α = 0.84–0.90).

##### Self-Schema Questionnaire

The Self-Schema Questionnaire (SSQ), adapted from the work of Markus (1977) and further refined by Andersen and Cyranowski (1994), was used to assess the presence, valence, and strength of self-schemas—that is, individuals’ cognitive generalizations about themselves. The version employed in this study consisted of 30 items, divided into positive and negative self-schema statements, rated on a 6-point Likert scale (1 = *Strongly Disagree* to 6 = *Strongly Agree*). Higher scores on positive schema items reflect a more adaptive and confident self-view, while higher scores on negative schema items indicate maladaptive or self-critical self-concepts. The SSQ has shown good internal consistency in Middle Eastern populations (Cronbach’s α = 0.82–0.88) (27).

##### General Health Questionnaire-28

The General Health Questionnaire-28 (GHQ-28), developed by Goldberg and Hillier (1979) (28), is a widely used screening tool for detecting psychological distress and minor psychiatric disorders in the general population. It consists of 28 items, divided into four subscales: *somatic symptoms*, *anxiety/insomnia*, *social dysfunction*, and *depression* (7 items each). Each item is scored on a 4-point Likert scale (0–3), yielding a total score between 0 and 84. Higher scores indicate greater psychological distress. A total score of ≥24 is commonly used as the cut-off value suggesting probable psychological morbidity. The GHQ-28 has been validated across multiple cultures, including Arabic-speaking populations, showing high reliability (Cronbach’s α = 0.89) and strong construct validity.

### Qualitative tools

The semi-structured interview guide, to explore from the participants perspective their lived experience of self-perception and identity reconstruction, and how they coped post conflict Mosul, was developed for this research. The questions asked included personal definitions of self-worth, whether or not they perceived a change in their self-image, and how they viewed this change as impacted by cultural and societal expectations.

### Data collection procedure

Data collection occurred throughout the month of May in 2025. Individuals interested in participating were identified and recruited by posting community announcements, coordinating with local health care facilities, and referrals from community leaders, and/or primary health care staff. Once potential participants were identified, they were approached by trained research assistants that explained the general purposes of the study, and the eligibility criteria, and the participation requirements. If the participant agreed to participate, they would then be screened for eligibility, and they would receive detailed information about the study.

Quantitative surveys were administered face-to-face by culturally competent, and fluent in Arabic, trained research assistants. The surveys were administered in the location most convenient and comfortable for each participant, i.e., their home, work place, or community center.

For the qualitative portion of the study, those participants that completed the quantitative survey, and agreed to participate further, were contacted and asked if they would be willing to participate in a one-on-one, in-depth interview. The interviews were audio recorded with prior participant consent, and were transcribed verbatim. Interviews generally took between 40–60 minutes to complete. Prior to collecting data, all participants were given a written information sheet detailing the objective(s) of the study, confidentiality procedures, and the participant's rights to withdraw from the study at any point in time; subsequently, written informed consent was acquired.

### Data analysis

The data collected via quantitative methods were evaluated by the use of SPSS (Version [28]) to generate information on the demographic and psychological attributes of the study participants utilizing descriptive statistics (i.e., mean, standard deviation & frequency).

Pearson’s Correlation Coefficient (r) was employed to examine the relationships between self-esteem, self-schema and mental health measures, while Multiple Linear Regression Analyses were performed to determine predictive variables of mental health status. In general, a p-value of ≤ 0.05 was used to determine whether or not there was a statistically significant relationship.

Additionally, 95% confidence intervals (95% CI) were created for both the correlation and regression coefficients generated during this investigation. For the confidence interval of Pearson’s r, the Fisher Z-Transform Method was utilized. Likewise, the confidence interval for the standardized regression coefficients (β) was calculated as β ± 1.96 * SE; where SE is the Standard Error of β. The Standard Error of β is derived from β/t.

Thematic Analysis was employed as the methodology to evaluate the data collected via qualitative methods. An inductive coding structure was developed to allow the researcher to iteratively identify themes within the transcripts. The researcher applied this coding structure in order to establish credibility of the findings from the study. Qualitative and quantitative data were triangulated to create a richer understanding of the study results.

### Ethics considerations

Ethics approval to conduct this work was obtained from the Research Ethics Committee, College of Nursing, University of Nineveh(Approval No. CCMRE-Nur-25-103). Similarly, we obtained prior to fieldwork, permission to conduct this study from relevant community leaders and health authorities in the locality. All of the participants received a written informed consent document prior to conducting to participate. If any of the participants found themselves in a position of ethical concern regarding the study or felt a psychological distress, the study authors indicated they would assist them with the protocols. The audio-recordings were kept in a locked cabinet and off-site each of the participants was provided a numerical code to allow continuous collection and validate confidentiality; neither of the authors had access to the codes, and there was no identifiable information on the transcripts. Participation was voluntary, with participants informed they could withdraw from the research at any time, and that any such withdrawal was made without issue and with no consequence. For a couple of participants who had reported acute high levels of psychological distress during their interviews, we provided them some information on local mental health services they could utilize.

## 3 Results

### 3.1 Participant characteristics

A total of 232 participants completed the study, yielding a response rate of 96.6%. The mean age was 31.4 years (SD = 9.7, range = 18–58 years), with females representing 54.3% (n = 126) of the sample. Most participants were married (60.8%, n = 141), and 48.3% (n = 112) held a university degree. Regarding employment status, 42.2% (n = 98) were employed, 28.4% (n = 66) were students, and 29.3% (n = 68) were unemployed. **Table 1** summarizes the socio-demographic characteristics.

**Table 1:** Socio-demographic characteristics of participants.

Variable	n (%) or mean ± SD
Age (years)	31.4 ± 9.7
Gender (Male/Female)	106 (45.7)/126 (54.3)
Marital Status	Single: 91 (39.2)/Married: 141 (60.8)
Education	Secondary: 57 (24.6)/Diploma: 63 (27.1)/University: 112 (48.3)
Employment	Employed: 98 (42.2)/Student: 66 (28.4)/Unemployed: 68 (29.3)

### 3.2 Self-esteem scores

The Rosenberg Self-Esteem Scale (RSES) scores ranged from 12 to 38, with a mean of 25.7 ± 5.9. Based on established cut-off values, 41.4% (n = 96) of participants had low self-esteem, 38.8% (n = 90) moderate self-esteem, and 19.8% (n = 46) high self-esteem.

### 3.3 Self-schema profiles

The Self-Schema Questionnaire (SSQ) revealed that participants had a mean positive self-schema score of 4.8 ± 1.1 (out of 6) and a mean negative self-schema score of 3.7 ± 1.4 (out of 6). Positive self-schemas were more prevalent in participants with higher education and stable employment, while negative self-schemas were significantly higher among unemployed and widowed/divorced participants (p < 0.05).

### 3.4 Mental health status

The General Health Questionnaire-28 (GHQ-28) indicated that 52.6% (n = 122) of participants had scores suggestive of possible psychological distress. The highest subscale mean was recorded for anxiety/insomnia (8.9 ± 4.2), followed by social dysfunction (8.4 ± 3.9), depression (7.6 ± 4.0), and somatic symptoms (7.2 ± 3.8).

### 3.5 Relationship between self-perception and mental health

Pearson Correlation Analysis revealed statistically significant relationships among self-perceptions and mental health outcomes. Positive correlations were found for self-esteem (r = .52, 95% CI [.42, .61] p < .01) and positive self-schemas (r = .48, 95% CI [.37,.57] p < .01) (**Table 2**). The results indicated that those who perceived themselves as having high levels of self-esteem and/or positive self schemas demonstrated enhanced mental health. Conversely, a negative correlation was observed for negative self-schemas (r = −.55, 95% CI [−.63,-.44] p < .01); indicating maladaptive self schemas are associated with increased psychological distress.

**Table 2:** Correlation coefficients (r) and 95% confidence intervals between self-perception variables and mental health (n = 232).

Variable Relationship	r	95% CI
Self-Esteem ↔ Mental Health	0.52	0.42–0.61
Positive Self-Schemas ↔ Mental Health	0.48	0.37–0.57
Negative Self-Schemas ↔ Mental Health	–0.55	–0.63 to –0.44

Computed using Fisher’s z-transformation (95% CI = z ± 1.96 × SE, where SE = 1/√(n–3)).

### 3.6 Predictors of mental health

Multiple regression analysis (**Table 3**) identified self-esteem (β = 0.37, 95% CI [0.26, 0.49], p < 0.001), negative self-schemas (β = –0.34, 95% CI [–0.45, –0.22], p < 0.001), and employment status (β = 0.19, 95% CI [0.04, 0.34], p = 0.014) as significant predictors of mental health scores, collectively explaining 46.2% of the variance (Adjusted R² = 0.462, F(5,226) = 34.1, p < 0.001).

**Table 3:** Multiple regression analysis predicting mental health scores.

Predictor	β	t	SE	95% CI	p-value
Self-Esteem	0.37	6.42	0.058	0.26–0.49	<0.001
Negative Self-Schemas	–0.34	–5.78	0.059	–0.45 to –0.22	<0.001
Employment Status	0.19	2.47	0.077	0.04–0.34	0.014
Age	–0.08	–1.36	0.059	–0.19–0.03	0.175
Gender	0.05	0.91	0.055	–0.06–0.16	0.364

### 3.7 Qualitative findings

Three primary themes emerged from thematic analysis of in-depth interview data.

1. Restoring Identity Post-Conflict: Participants stated how they attempted to establish or restore their self-worth after experiencing displacement; loss of livelihoods; and/or significant financial hardship.
2. The Influence of Cultural Pressures on Self-Schemata: Social expectations surrounding personal identity (e.g., perceived honor; reputation; gender) significantly impacted participants' self-esteem, and self-perceptions of themselves.
3. Fostering Resilience via Neighborhood Solidarity: Participants reported that social support networks (family, religion, neighborhoods) contributed to maintaining a positive self-image for them during periods of challenge.

Illustrative quote:

*“Even after everything we lost, I try to remind myself that I am still valuable. My family depends on me, and that gives me strength.”*

(Female, 34 years)

## 4 Discussion

From a theoretical perspective, this study is grounded in Cognitive Theory, which emphasizes the role of cognitive processes—such as beliefs, attitudes, and self-schemas—in shaping emotional and behavioral responses. According to Beck’s Cognitive Theory of Depression, individuals interpret experiences through cognitive frameworks that influence their perceptions of self, others, and the world. Maladaptive cognitions, such as negative self-schemas or distorted self-beliefs, contribute to emotional distress and the onset of psychological disorders. Conversely, adaptive cognitions and positive self-perceptions foster emotional regulation and resilience. Within the context of post-conflict Mosul, where individuals have endured chronic stress, trauma, and social disruption, understanding how these cognitive structures influence mental health becomes essential. Thus, exploring self-perception through the lens of Cognitive Theory provides a foundation for identifying both risk and protective factors that may inform culturally appropriate psychological interventions.

This study examined the relationship between self-perception—operationalized as self-esteem and self-schemas—and mental health among adults in the city of Mosul, Iraq, using a mixed-methods approach. The findings demonstrate a significant association between positive self-perception and better mental health outcomes, while negative self-schemas were strongly linked to psychological distress. These results highlight the central role of self-perception as both a protective and risk factor for mental health in post-conflict settings.

### 4.1 Self-esteem and mental health

The proportion of participants with low self-esteem in this study (41.4%) was comparable to findings from Baghdad (39%) and higher than results reported in Jordan (32%) and Egypt (28%). This suggests that post-conflict environments, such as Mosul, may exacerbate self-perception difficulties and psychological distress (29, 30). In the context of Mosul, prolonged exposure to conflict, displacement, and economic instability may have undermined individuals’ sense of self-worth, thereby exacerbating mental health vulnerabilities. Qualitative data further supported this, as participants frequently linked feelings of diminished value to unemployment, loss of social roles, and perceived inability to meet cultural expectations.

### 4.2 Self-schemas as cognitive filters

The study found that positive self-schemas were associated with improved mental health, whereas negative self-schemas were predictive of psychological distress, consistent with cognitive theory (31). Negative self-schemas—formed through repeated exposure to adverse experiences—likely serve as cognitive filters that reinforce pessimistic self-views and maladaptive coping. In post-conflict Mosul, the disruption of personal and social identity due to war, as reflected in participants’ narratives, may have contributed to the persistence of maladaptive self-schemas. Importantly, these findings resonate with prior research in trauma-affected populations, which underscores the difficulty of altering entrenched negative beliefs without targeted psychosocial interventions (3, 32).

### 4.3 Cultural context and social expectations

A key contribution of this study lies in its exploration of how cultural and societal norms in Mosul shape self-perception. The qualitative themes revealed that cultural constructs related to family honor, reputation, and gender roles significantly influenced both self-esteem and self-schemas. While these values can promote resilience through strong community ties, they may also impose rigid expectations that amplify self-criticism when individuals fall short. This dual role of cultural norms is consistent with findings from Middle Eastern mental health research, where collectivist values serve as both a source of social support and a potential source of psychological pressure (1, 33).

### 4.4 Resilience and recovery

Although there were a number of challenges that many participants reported as contributing to their ability to draw upon sources of community solidarity, religious faith and family support; these factors ultimately served to provide the basis for maintaining, or reconstructing, a positive self-perception—consistent with the body of literature on resilience, that emphasizes the role of social connection and meaning-making in protecting against the negative psychological consequences associated with post-conflict recovery (34, 35). Therefore, it is suggested that when designing mental health intervention programs in Mosul, not only will programs need to be designed to intervene in maladaptive self-schemas, but also they will need to incorporate culturally relevant and accessible sources of resilience.

### 4.5 Implications for practice and policy

The high predictive power of self-esteem and negative self-schemas in predicting mental health outcomes in this research supports the use of interventions focused on self-esteem and negative self-schemas. A cognitive-behavioral approach to treatment that focuses on changing maladaptive beliefs, along with culturally appropriate psycho-educational programs, may have special utility as an intervention strategy. In terms of public policy, incorporating self-perception assessments in routine mental health screenings in primary care and in community settings would likely lead to earlier identification of persons who are at risk of poor mental health. Community based initiatives to restore economic opportunities and social roles may also have a positive impact on both self-perception and mental health.

### 4.6 Limitations and future research

The research had several constraints. The cross-sectional nature of the data collection does not allow for making conclusions about cause-and-effect relationships, and as such the data collected through self-reporting may have been biased based on social desirability (i.e., people reporting things they think others want them to report rather than what is actually true) in light of the mental health stigma that exists in the culture from which the sample is derived; this research was also limited to only those living in Mosul; therefore it is possible that the findings do not provide an accurate representation of displaced persons or rural communities. Future longitudinal designs will help examine the evolution of self perception and its relationship to post conflict recovery processes; additional longitudinal study would also provide the opportunity to evaluate the effectiveness of self perception focused interventions for improving the mental health outcomes for displaced persons in post conflict areas

### 4.7 Conclusion

The findings support the idea that the self-perceived (self-esteem & self-schema) is a major determinant of adult mental health in Mosul, Iraq; therefore interventions that improve positive perceptions of the self and reduce negative or maladaptive schemas can be used to increase psychological resiliency and overall well-being for adults living in areas experiencing conflict

## Data Availability

The raw data supporting the conclusions of this article will be made available by the authors, without undue reservation.
